# Association of Participation in the Collaborative Care Team With Behavioral and Psychological Symptoms of Dementia and Caregiver Distress

**DOI:** 10.1002/brb3.71694

**Published:** 2026-08-17

**Authors:** Yu‐Hsuan Hung, Wen‐Fu Wang, Ming‐Che Chang, Yu‐Chun Tung, Kai‐Ming Jhang

**Affiliations:** ^1^ Department of Ophthalmology Show Chwan Memorial Hospital Changhua Taiwan; ^2^ Neurological Institute Changhua Christian Hospital Changhua Taiwan; ^3^ Department of Nuclear Medicine Changhua Christian Hospital Changhua Taiwan; ^4^ Department of Pharmacy Taichung Veterans General Hospital Taichung Taiwan

**Keywords:** behavioral and psychological symptoms of dementia (BPSD), dementia collaborative care, Disease‐Specific Care (DSC) certification program, Joint of Commission of Taiwan (JCT), neuropsychiatric symptoms

## Abstract

**Introduction:**

The management of behavioral and psychological symptoms associated with dementia (BPSD) was challenging for the physicians and their caregivers, which placing a great burden on their caregivers, families, and communities. Our study aimed to explored the factors associated with changes in BPSD severity and caregiver distress.

**Methods:**

This retrospective cohort study examined 738 community‐dwelling individuals who were newly diagnosed with dementia and had BPSD at baseline. Participants were categorized based on a case manager–centered collaborative model that evaluated unmet needs and offered appropriate interventions to patients and caregivers. A generalized linear model with backward selection identified factors linked to changes in total NPI and NPI Distress scores one year after diagnosis.

**Results:**

Participants dismissed during follow‐up (compared with those with continuous follow‐up, *β* = 3.2, 95% CI = 0.83–5.5, *p* = 0.008) and participants cared for by a sole informal caregiver (compared with ADL‐independent participants whose caregivers only provide companionship, *β* = 3.9, 95% CI = 1.2–6.7, *p* = 0.006) were significantly associated with higher NPI total scores. Male sex (*β* = –3.0, 95% CI = –4.9, –1.0, *p* = 0.003), and using assistive devices and home modifications (*β* = –3.0, 95% CI = –6.0, –0.01, *p* = 0.05) were protective factors. Similar patterns emerged for caregiver distress, with follow‐up discontinuation (*β* = 1.7, 95% CI = 0.18, 3.2, *p* = 0.029), using respite care (*β* = 4.4, 95% CI = 0.00, 8.7, *p* = 0.05), and participants cared by a sole informal caregiver (*β* = 2.7, 95% CI = 0.95, 4.4, *p* = 0.003) increasing distress.

**Conclusions:**

The case manager–centered dementia collaborative care model was associated with reduced short‐term BPSD severity and caregiver distress, with sex, care mode, and social resource use as predictive factors. Integrating collaborative care models in long‐term care healthcare systems is recommended to better support dementia patients and caregivers.

## Graphical abstract

The case manager–centered dementia collaborative care model was associated with reduced short‐term BPSD severity and caregiver distress. Integrating collaborative care models in long‐term care healthcare systems is recommended to better support dementia patients and caregivers.

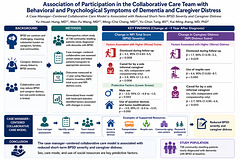

## 1 Introduction

In Taiwan, dementia is estimated to affect more than 3.4% of the population between 65‐ and 69‐year age, and its prevalence continues to rise due to the rapid aging of the population (Ministry of Health and Welfare 2014). Taking care of patients with dementia places a great burden on their caregivers, families, and communities (Chen and Fu 2020; Ministry of Health and Welfare 2014). The most well‐known clinical manifestations of dementia are cognitive impairment and memory loss; however, the management of behavioral and psychological symptoms associated with dementia (BPSD) was more challenging for not only the physicians but also their caregivers (Chen et al. 2021).

The Neuropsychiatric Inventory (NPI) was used to assess the severity of BPSD and to divide the complicated symptoms into 12 domains: delusions, hallucinations, dysphoria, anxiety, agitation/aggression, euphoria, disinhibition, irritability/lability, apathy, aberrant motor activity, nighttime behavior, and appetite disturbance (Cummings et al. 1994). In a large study that included more than 10,000 individuals living in long‐term care facilities, aberrant motor behavior, agitation, and irritability were the most common symptoms among the different types of dementia (Schwertner et al. 2022). Moreover, a previous study mentioned that the prevalence of BPSD characteristics ranged from 4% (elation and mania) to 32% (apathy) in community‐dwelling patients with dementia, and prioritizing these symptoms when formulating long‐term care programs for these patients is recommended (Kwon and Lee 2021).

Multiple factors, including patient, caregiver, and environmental factors may contribute to the deterioration of BPSD (Kales et al. 2015). Women with mild Alzheimer's disease (AD) tended to suffer more frequently from BPSD than men, and old age was also perceived as a risk factor for BPSD (Boccardi et al. 2017; Colombo et al. 2018). Another study found that aggravated BPSD was associated with increased dementia severity among patients residing at home (Wan et al. 2021).

While a high proportion of patients with dementia are community‐dwelling, caregiver factors affecting BPSD should be identified (Clark et al. 2018). Studies have shown that caregivers with a positive perspective and lower expressed emotions during the care process were associated with the alleviation of BPSD (Baharudin et al. 2019; Wan et al. 2021). The frequency of some neuropsychiatric symptoms (NPS) showed a significant correlation of caregivers' burden (Lin et al. 2022).

To relieve the burden on caregivers, the Taiwanese government established the Long‐Term Care Plan 2.0, which includes people with dementia over the age of 50 (Hsu and Chen 2019). Several long‐term care services have been implemented, including personal and professional services, transportation services, assistive devices, respite care services, and community‐aging care centers (Huang et al. 2021; Jhang et al. 2019; Lai et al. 2022). A series of dementia care services, such as dementia integrated care centers, implement dementia case management to create individualized care plans. Few studies have investigated the association between these services and severity of BPSD or caregiver stress.

The predictors of BPSD progression and caregiver distress cannot be ignored. While family caregivers usually experience more distress caused by BPSD than formal caregivers in nursing homes, it is necessary to investigate the risk factors and provide individualized care plans to fit their needs (Jhang et al. 2019; Jhang et al. 2023). Previous studies found that withdrawal from the dementia collaborative care model was a risk factor for BPSD deterioration, and the benefits of interventions provided by case‐based management programs for patients with BPSD were also mentioned in some studies (Callahan et al. 2006; Hung et al. 2023). Considering that few studies discussed the efficacy of intervention by case managers on both BPSD and caregivers' distress, the present study aimed to explore the factors associated with change in NPI and NPI distress (NPI‐D) scores. The baseline characteristics of the patients and caregivers, utilization of social resources, and joining status of the collaborative care model were recorded.

## 2 Methods

### 2.1 Data Source and Study Population

**FIGURE 1:**
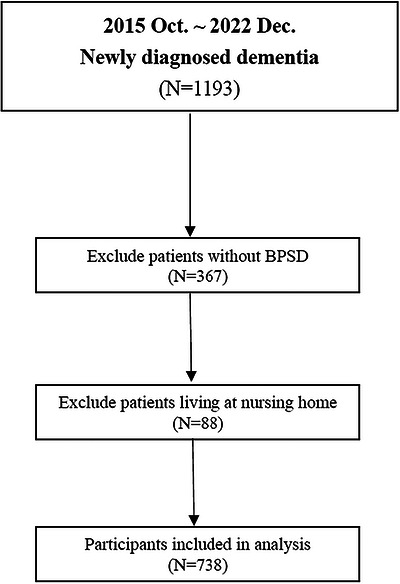
Participants flow diagram.

This retrospective cohort study was conducted at Changhua Christian Hospital (CCH) in Central Taiwan from October 2015 to December 2022. We enrolled patients who were newly diagnosed with dementia. Both patients and their caregivers underwent initial assessments, including cognitive function, BPSD, living status and care modes, caring problems, caregiver burden, and preference for utilizing long‐term care resources. Patients presenting with any neuropsychiatric symptoms (NPS) and still living in the community were selected for analysis. Figure 1 shows the participant flow diagram.

This study was approved by the Institutional Review Board of the CCH (CCH IRB 230310). The requirement for informed consent was waived by the Institutional Review Board of Changhua Christian Hospital because all data required in this study (including both patient‐ and caregiver‐reported data such as caregiver distress) were extracted from electronic charts after deleting personalized information.

### 2.2 Case Manager–Centered Collaborative Model

Our dementia collaborative model is comprised of various professionals, including physicians, psychologists, social workers, dieticians, occupational therapists, pharmacists, and nursing case managers. The attending physicians invited the patients and their carers to join the model. Every outpatient visit with the International Classification of Disease (ICD) coding of dementia was automatically retrieved from the dementia case management system, which is an informative system established in the medical recording system of CCH (Chen et al. 2024). Case managers sent messages to attending specialists for patient referral within 3 months of the first ICD coding for dementia. Patients and caregivers joined the collaborative care model after providing informed consent from attending specialists. After joining the care model, face‐to‐face evaluations were conducted every 6 months to identify unmet care needs and provide relevant interventions (Jhang et al. 2019; Jhang et al. 2023). Among these team members, nursing case managers play an important role in keeping contact with the patients and caregivers. Case managers contacted participants via telephone every month to confirm whether their needs were properly met. The care team passed the Disease‐Specific Care (DSC) Certification Program for Dementia held by the Joint Commission of Taiwan (JCT) in July 2021.

### 2.3 Measurement of BPSD and Caregiver Distress

Patient cognition and BPSD status were evaluated annually by clinical psychologists at our hospital for patients with cognitive impairment, regardless of whether they participated in the care model. The Neuropsychiatric Inventory (NPI) was used to assess behavioral and psychological disturbances in patients with dementia (Cummings et al. 1994). These NPS included delusions, hallucinations, dysphoria, anxiety, agitation/aggression, euphoria, disinhibition, irritability/lability, apathy, aberrant motor activity, night‐time behavior, and appetite disturbance. Frequency (1 = rarely, less than once per week; 2 = sometimes, about once per week; 3 = often, several times per week; and 4 = very often, once or more per day) and cross‐severity (1 = mild, 2 = moderate, 3 = severe) were calculated for each symptom domain, and the sum of the 12 symptoms was the total NPI score, ranging from 0 to 144 points. Caregiver distress (0 = no distress, 1 = minimal distress, 2 = mild distress, 3 = moderate distress, 4 = severe distress, and 5 = very severe distress) was rated for each symptom domain, and the total distress score was the sum of each domain. The NPI was performed at the initial assessment and the second evaluation approximately one year later. Patients without any NPS during the first interview were excluded. According to the joining status of the collaborative care model at the two NPI‐assessed time points, participants were categorized into continuous care, withdrawal, dismissal from follow‐up, and midway‐joining groups. Patients who joined the care model at both NPI assessment times were assigned to the continuous care group. Among continuous care group, patients and their caregiver received face‐to‐face interview every 6 months and telephone contact every month by case managers between NPI assessment period. Patients who participated in the model at the first NPI interview but refused or could not cooperate with the following contact were defined as withdrawn from the care model. Patients not entering the care model at both NPI assessments were categorized as dismissed from follow‐up. Finally, the midway‐joining group included patients recorded as entering the model at the second NPI, but not at the initial assessment.

### 2.4 Measurement of Patient Feature

The demographic characteristics of the patients, including age, sex, severity of dementia measured by the clinical dementia rating scale sum of boxes (CDR‐SOB), subtype of dementia, and walking ability, were collected during the initial assessment.

Dementia subtypes were classified and diagnosed according to different guidelines. AD was corresponds to the National Institute on Aging‐Alzheimer's Association (NIA‐AA) (Albert et al. 2011; McKhann et al. 2011) whereas the diagnostic criteria for vascular cognitive impairment (VCI) are from the International Society for Vascular Behavioral and Cognitive Disorders (VASCOG) (Sachdev et al. 2014). The diagnosis of Parkinson's disease dementia (PDD) or DLB was corresponding to the Movement Disorder Society‐Task force criteria and the fourth consensus report of the DLB Consortium (Emre et al. 2007; McKeith et al. 2017). Lewy body disorders (LBD) include PDD and DLB. Patients diagnosed with both AD and VCI were classified as mixed type.

### 2.5 Measurement of Caregiver Feature

Data on caregivers’ age, education level, relationship with patients, cohabitation status, care mode, and usage of social resources status at baseline NPI evaluation were recorded.

According to the payment categorization in the Long‐Term Care Plan 2.0, in Taiwan, long‐term care resources are divided into four items: personal and professional care, transportation services, assistive devices, in‐house barrier‐free environment modification, and respite care (Jhang et al. 2019; Joint Commission of Taiwan 2018). Personal and professional care included in‐home and community services such as home personal care or daycare services, home nursing, and home rehabilitation services. Transportation services refer to shuttling from homes to hospitals. The assistive device service refers to the devices suggested by therapists. Respite care was often provided by personal care attendants to let caregivers rest for hours to days. Additionally, community aging care centers provide congregate meals and general medical information. Exercise and cognitive training programs were also promoted for healthy, pre‐frail, and frail elderly individuals living in the community.

### 2.6 Statistical Analyses

R software (R Foundation for Statistical Computing) was used to generate all data in this study. Independent variables included patient and caregiver characteristics. The dependent variables were changes in total caregiver distress or NPI scores, which were defined as the difference between the first and second NPI assessments. Differences between categorical data were calculated using the chi‐square test and Fisher's exact test. Numerical data were analyzed using Analysis of Variance (ANOVA) test. A generalized linear model with backward selection was used to test the factors associated with changes in the total NPI score and total caregiver distress with adjustment of baseline NPI or caregiver distress scores. Ten cases with missing caregiver distress scores were excluded from the generalized linear model analysis. Statistical significance was determined when the *p* value was less than 0.05.

## 3 Results

Table 1 shows the demographic characteristics of the participants.

**TABLE 1:** Demographic characteristics of patients and caregivers.

	Continuous care group, *N* = 471	Withdraw group, *N* = 31	Dismissed group, *N* = 157	midway‐joining group, *N* = 79	*p*‐value
**Patient's factor**					
Diagnosis, type of dementia					
AD	284 (60%)	15 (48%)	92 (59%)	43 (54%)	0.246
VCI	76 (16%)	6 (19%)	17 (11%)	14 (18%)	
Mixed^a^	12 (2.5%)	0 (0%)	9 (5.7%)	1 (1.3%)	
LBD	42 (8.9%)	3 (9.7%)	19 (12%)	7 (8.9%)	
others	57 (12%)	7 (23%)	20 (13%)	14 (18%)	
Age	78.2 (8.1)	80.6 (10.3)	78.7 (8.1)	75.7 (7.4)	0.003
Gender: female	302 (64%)	24 (77%)	89 (57%)	49 (62%)	0.13
Gender: male	169 (36%)	7 (23%)	68 (43%)	30 (38%)	
CDR‐SOB	5.3 (3.9%)	6.1 (4.1)	5.5 (3.9)	4.2 (3.6)	0.015
Walking ability					
Free	304 (65%)	21 (68%)	89 (57%)	48 (61%)	0.631
Assisted device	136 (29%)	6 (19%)	53 (34%)	24 (30%)	
Wheelchair	30 (6.4%)	4 (13%)	15 (9.6%)	7 (8.9%)	
Bedridden	1 (0.2%)	0 (0%)	0 (0%)	0 (0%)	
Education (year)	5.4 (4.4)	4.5 (4.3)	5.4 (4.7)	6.4 (5.0)	0.2
**Caregiver's factor**					
Relationship					
Spouse	140 (30%)	7 (23%)	46 (29%)	30 (38%)	
Offspring	266 (56%)	17 (55%)	89 (57%)	36 (46%)	0.473
Others	65 (14%)	7 (23%)	22 (14%)	13 (16%)	
Age^b^	58.2 (14.0)	56.8 (12.5)	58.8 (13.6)	60.3 (14.2)	0.6
Cohabitants					
Living alone	9 (1.9%)	1 (3.2%)	4 (2.5%)	0 (0%)	
Spouse	111 (24%)	6 (19%)	36 (23%)	29 (37%)	
Spouse and children	163 (35%)	11 (35%)	54 (34%)	20 (25%)	0.146
Children	148 (31%)	13 (42%)	57 (36%)	25 (32%)	
Others	40 (8.5%)	0 (0%)	6 (3.8%)	5 (6.3%)	
Care mode^c^					
0	179 (38%)	9 (29%)	54 (34%)	30 (38%)	0.048
1	154 (33%)	9 (29%)	63 (40%)	17 (22%)	
2	7 (1.5%)	1 (3.2%)	2 (1.3%)	0 (0%)	
3	46 (9.8%)	2 (6.5%)	20 (13%)	12 (15%)	
4	85 (18%)	10 (32%)	18 (11%)	20 (25%)	
Usage of long‐term care resources					
Personal and professional care	79 (17%)	7 (23%)	27 (17%)	10 (13%)	0.6
Transportation(shuttle)	34 (7.2%)	0 (0%)	8 (5.1%)	4 (5.1%)	0.4
Assisted devices	47 (10.0%)	5 (16%)	24 (15%)	10 (13%)	0.2
Respite care	7 (1.5%)	0 (0%)	3 (1.9%)	4 (5.1%)	0.2
Community aging care centers	23 (4.9%)	2 (6.5%)	4 (2.5%)	4 (5.1%)	0.5

^a^ “Mixed type” indicates mix AD and VCI.

^b^ 1 missing data.

^c^The care mode indicates that caregivers care for people living with dementia most of the time.

Mode 0 = ADL‐independent, caregivers only accompanied the subject; Mode 1 = care by a sole informal caregiver; Mode 2 = care by more than two caregivers (including foreign care workers); Mode 3 = care at different children's homes alternately; Mode 4 = care by sole foreign care workers.

Abbreviations: AD, Alzheimer's disease; CDR‐SOB, Clinical Dementia Rating Scale Sum of Boxes; LBD, Lewy body disease; NPI, neuropsychiatric inventory; VCI, vascular cognitive impairment.

A total of 738 patients were enrolled in this study (Figure 1). The mean age of the patients was 78.1 years old, with the highest mean age (80.6 years old) observed in the withdrawal group. Female was the predominant sex in all groups. Alzheimer's disease (59%) was the most common type of dementia in all groups. Regarding dementia severity, the highest CDR‐SOB score was noted in the withdrawal group (6.1), whereas the lowest was in the midway‐joining group (4.2). Concerning caregiver factors, the overall mean age was 58.5 years old, and most were the patients' offspring (55%). The majority of the care modes were Mode 1 (care by an informal caregiver, 37%) and Mode 2 (care by more than two caregivers, 33%). Among the five long‐term care resources, personal and professional care (17%) were the most commonly utilized.

Table 2 indicates the changes in the NPI and caregivers’ distress scores according to the status of joining the collaborative care model. The time interval between the first and second assessments was approximately 1.3 years. The highest NPI score was initially present in the withdrawal group (13.4), while an elevation in the NPI score was only found in the discontinuation group (1.9). As for caregivers' distress scores, the withdrawal group still had the highest score (9.2), but increased levels of distress scores were noted in both the withdrawal group (0.7) and the dismissal group (1.4).

**TABLE 2:** Changes of total NPI score and caregivers’ distress score according to joining status of collaborative care model.

	Continuous care group, *N* = 471	Withdraw group, *N* = 31	Dismissed group, *N* = 157	midway‐joining group, *N* = 79	*p*‐value
Time interval (years)	1.3 (0.8)	1.2 (0.4)	1.3 (0.7)	1.3 (0.7)	0.8
NPI score					
Initial NPI score	11.5 (12.8)	13.4 (10.7)	10.9 (11.3)	11.0 (12.0)	0.4
Followed NPI score	10.2 (11.9)	12.5 (17.2)	12.8 (15.8)	10.4 (14.2)	0.8
NPI score change	−1.3 (15.0)	−0.9 (17.4)	1.9 (15.2)	−0.6 (17.1)	0.3
Caregivers’ distress score					
Initial distress score^a^	7.7 (8.0)	9.2 (8.5)	7.2 (8.3)	7.9 (7.8)	0.5
Followed distress score^b^	7.3 (7.8)	9.8 (10.5)	8.6 (10.1)	7.3 (8.5)	0.7
Distress score change^c^	−0.4 (9.5)	0.7 (11.8)	1.4 (11.6)	−0.6 (9.2)	0.4

aSix missing data.

^b^Five missing data.

^c^Ten missing data.

Table 3 shows the factors associated with changes in total NPI scores using generalized linear model with backward stepwise selection.

**TABLE 3:** Generalized Linear Model with backward stepwise selection to predict factors associated with change in NPI total score.

	*β* value	95% CI	*p*‐value
**Patient's factor**			
Joining status	Ref: Continuous care		
Withdraw	1.8	[–2.9; 6.4]	0.5
Dismissed	3.2	[0.83; 5.5]	0.008
Midway‐joining	0.99	[–2.1; 4.1]	0.5
Gender—female	Ref	—	—
Gender—male	−3.0	[–4.9; –1.0]	0.003
Baseline NPI total scores	−0.72	[–0.80; –0.64]	<0.001
CDR‐SOB	0.25	[–0.02; 0.52]	0.065
**Caregiver's factor**			
Usage of long‐term care resources	Ref: without using		
Personal and professional care	2.3	[–0.34; 4.9]	0.088
Respite care	6.5	[–0.35; 13]	0.063
Assisted devices and home modification	−3.0	[–6.0; –0.01]	0.050
Care mode	Ref: 0		
1	3.9	[1.2; 6.7]	0.006
2	1.9	[–0.94; 4.7]	0.2
3	0.43	[–7.8; 8.6]	>0.9
4	0.39	[–3.5; 4.3]	0.8

The dismissed from follow‐up group was significantly associated with worse NPI total scores when compared with continuous care group (*β* = 3.2, 95% CI = 0.83, 5.5, *p* = 0.008). Participants cared by a sole informal caregiver was associated with increased NPI total scores when compared with those ADL independent (*β* = 3.9, 95% CI = 1.2, 6.7, *p* = 0.006). Adversely, male sex (*β* = –3.0, 95% CI = –4.9, –1.0, *p* = 0.003) and use of assisted devices and home modification (*β* = –3.0, 95% CI = –6.0, –0.01, *p* = 0.05) were protective factors.

Table 4 shows the linear regression with a backward stepwise selection model to identify factors related to changes in caregivers' total distress scores.

**TABLE 4:** Generalized linear model with backward stepwise selection to predict factors associated with changes of caregivers' distress total score.

	*β* value	95% CI	*p*‐value
**Patient's factor**			
Joining status	Ref: Continuous care		
Withdraw	2.4	[–0.59; 5.4]	0.12
Dismissed	1.7	[0.18; 3.2]	0.029
Midway‐joining	0.25	[–1.8; 2.3]	0.8
Gender—female	Ref	—	—
Gender—male	−1.1	[–2.4; 0.27]	0.12
Baseline caregivers' distress total score	−0.73	[–0.81; –0.66]	<0.001
Education (year)	−0.11	[–0.25; 0.03]	0.14
**Caregiver's factor**			
Usage of long‐term care resources	Ref: without using		
Respite care	4.4	[0.00; 8.7]	0.050
Assisted devices and home modification	−1.8	[–3.7; 0.09]	0.062
Care mode	Ref: 0		
1	2.7	[0.95; 4.4]	0.003
2	1.7	[–0.09; 3.5]	0.062
3	0.06	[–5.2; 5.3]	>0.9
4	0.52	[–1.8; 2.9]	0.7

Both dismiss of follow‐up group (*β* = 1.7, 95% CI = 0.18, 3.2, *p* = 0.029), and use of respite care (*β* = 4.4, 95% CI = 0.00, 8.7, *p* = 0.05), and participants cared by a sole informal caregiver (*β* = 2.7, 95% CI = 0.95, 4.4, *p* = 0.003) were risk factors for increasing caregivers' distress.

## 4 Discussion

Our study showed that maintaining care in a dementia collaborative model was related to better BPSD severity and reduced caregiver distress among community‐dwelling individuals newly diagnosed with dementia who had BPSD at baseline. Compared with ADL‐independent participants, people with dementia cared for by a sole informal caregiver were associated with worsening BPSD. However, usage of assistive device and home modification services were found to be associated with decreased NPI total scores.

The highlight of this study is that different joining statuses in our collaborative model was associated with changes in both BPSD severity and caregiver distress. In our collaborative model, case managers are responsible for connections between patients, caregivers, and professionals such as physicians and social workers. In a randomized clinical trial, collaborative care provided recommendations for treatment with cholinesterase inhibitors, education on communication and coping skills for caregivers, and legal and financial advice (Callahan et al. 2006). These interventions from case manager interventions was associated with better 1‐year BPSD trajectories and a reduction in caregiver stress. Another study from the UCLA Alzheimer's and Dementia Care Program demonstrated clinical benefits for patients' behavioral symptoms and caregiver burden after year‐long interventions from nurse practitioners (NP) and Dementia Care Managers (DCMs) (Reuben et al. 2019). The interventions that care models provided were similar among previous studies, and the effectiveness of the case manager–centered collaborative care model on the patients’ BPSD and caregivers was also studied (Frost et al. 2020; Hung et al. 2023).

In this study, the dismissed group was associated with worse changes in NPI scores and more severe distress among caregivers. This could be attributed to the lack of follow‐up and adjustment for medication usage, caregiver education, caregiver support groups, and referral to dementia care services, although the aforementioned items were all included in our care programs. However, although the highest NPS and caregiver distress scores were initially presented in the withdrawal group, the scores did not significantly deteriorate as the dismissed group did, which may imply that even short‐term participation in the care model could benefit both patients and caregivers.

Male sex was associated with decreased NPI scores, but there was no statistically significant influence on caregiver distress. A retrospective cohort study reported more female patients in the severe BPSD group than in the mild BPSD group (Chang et al. 2020). In a Taiwanese study that focused on patients with AD, it was found that male patients had a longer education duration and higher MMSE scores than female patients, while female sex was related to specific BPSD such as delusion and disinhibition (Hsieh et al. 2020). A meta‐analysis also showed that female sex was more strongly associated with depressive symptoms, psychotic symptoms, and aberrant motor behavior. However, male sex was only associated with more severe apathy (Eikelboom et al. 2022). Consistent with previous studies, the present study evaluated the progression of NPS and found that BPSD in male patients was less likely to worsen.

The present results show that care mode may associate with NPI score changes. People with dementia cared by one informal caregiver were associate with increased NPI score and caregiver distress when compared with those who still ADL independent, and caregivers only accompanied the subject. Caregiving conditions may significantly influence the prognosis and clinical outcomes of patients with dementia (Norton et al. 2009). Previous studies reported that caregivers who were younger, less educated, more depressed, more burdened, or spent more hours per week giving care reported more neuropsychiatric symptoms of dementia in care recipients (Sink et al. 2006). Another study also found that female caregivers, presenting high levels of burden, a moderate degree of depressive symptoms, and using active management strategies and criticism management strategies tend to report higher severity of neuropsychiatric symptoms (Delfino et al. 2018 Aug). BPSD was perceived as a major predictor of caregiver distress among other contributing factors associated with caregiver distress (Wang et al. 2015). It is not surprising that caregivers who care for people with dementia alone spend most of their time caring for the patients and bear the highest caregiving burden. They may therefore observe more BPSD than caregivers using other care modes.

The choice of care service was also associated with changes in NPI scores and caregiver distress. A study from Taiwan showed that caregivers of patients with dementia could benefit from a combination of multiple long‐term services (Chan et al. 2022). Another study analyzed the usage pattern of long‐term care services and found that patients with mild dependency tended to use community support services, whereas patients with moderate to severe dependency were more likely to choose home care services (Wang et al. 2021). Our results also showed that utilization of assistive devices and home modification were protective factors, whereas respite care was a risk factor for the deterioration of BPSD distress scores. This may be partially explained by the content of second version of the long‐term care policy. Under the current policy, users and families can choose what they would like to use from a menu of care services, including home and daycare services, home nursing, rehabilitation, and nutrition services (Chen and Fu 2020). Nevertheless, users face difficulty distributing their budgeted funds in the category of professional and personal care services. Moreover, the service term is clearly defined and charged into the new payment system, which could lead to a greater economic or mental burden on family caregivers (Hsu and Chen 2019). However, it may take time to re‐evaluate the long‐term effects of this service and its influence on patients and their caregivers. Further studies are required to address this issue.

The strength of this study is the variety of contributing factors, such as the joining status of the collaborative care model, caregiver features, and utilization of long‐term care sources. Although few studies have investigated the influence of case management on BPSD and caregiver distress, the present study also considered the joint status of case management.

### 4.1 Limitations

Our study has some limitations. First, this was a single‐center study, and the results may be difficult to generalize to other hospitals. Second, this was an observational study. Participation in the collaborative care model was not randomized, and a selection bias may exist. Because the engagement status in the collaborative care model was determined during the follow‐up period, it may reflect patient and caregiver characteristics, motivation, caregiver capacity, access to care, family support, socioeconomic background, or early clinical course. The most likely reason for people with dementia being in the dismissed group was that caregivers refused interviews with case managers. Caregivers’ attitudes toward care teams may influence their care and therefore interfere with BPSD severity. The observed associations in the present study may be influenced by residual confounding and selection bias. However, this study included several caregiver characteristics in the analysis and attempted to minimize this effect. Third, the statistical analysis relied on change scores and stepwise linear regression, which may have potential bias since the participation status was not randomized and not a fixed baseline exposure. These issues limit causal interpretation. Fourth, this study only enrolled patients within 3 months of their first ICD code. However, the presentation of NPS fluctuates during dementia progression. The current findings only identified risk factors for short‐term changes in BPSD severity and caregiver distress. Long‐term follow‐up is needed to provide more precise results regarding the effectiveness of case manager based care model.

## 5 Conclusions

The case manager–centered dementia collaborative care model was associated with lower short‐term BPSD severity and caregiver distress among community‐dwelling individuals newly diagnosed with dementia who had BPSD at baseline. Improvement of NPI scores one year after the diagnosis of dementia was also observed in patients using assisted devices and home modification, while female sex and participants only cared by single informal caregiver were associated with worse BPSD. Long‐term care policy and healthcare systems should consider introducing collaborative care models and more case managers for patients and their families.

## Author Contributions

**Yu‐Hsuan Hung**: conceptualization, investigation, Writing – original draft. **Ming‐Che Chang**: methodology, validation, formal analysis, writing – review and editing. **Wen‐Fu Wang**: writing – review and editing, methodology, formal analysis, validation. **Yu‐Chun Tung**: methodology, formal analysis. **Kai‐Ming Jhang**: methodology, formal analysis, supervision, project administration, writing – review and editing, writing – original draft.

## Funding

This study was supported by CCH Grant 113‐CCH‐IRP‐011.

## Ethics Statement

The requirement for informed consent was waived by the Institutional Review Board of Changhua Christian Hospital because all data (including both patient‐ and caregiver‐reported data such as caregiver distress) required in this study were extracted from electronic charts after deleting identifiable information. All procedures were conducted in accordance with the Declaration of Helsinki. The corresponding author had full access to the study's data and is responsible for its integrity and analysis. The data supporting the findings of this study are available from the corresponding author upon reasonable request. This article adheres to the guidelines of the Strengthening the Reporting of Observational Studies in Epidemiology

## Conflicts of Interest

The authors have no conflicts.

## Data Availability

The data that support the findings of this study are available on request from the corresponding author. The data are not publicly available due to privacy or ethical restrictions.
